# Genome Sequence of *Pandoraea* sp. ISTKB, a Lignin-Degrading Betaproteobacterium, Isolated from Rhizospheric Soil

**DOI:** 10.1128/genomeA.01240-16

**Published:** 2016-11-03

**Authors:** Madan Kumar, Rajesh Kumar Gazara, Sandhya Verma, Manish Kumar, Praveen Kumar Verma, Indu Shekhar Thakur

**Affiliations:** a School of Environmental Sciences, Jawaharlal Nehru University, New Delhi, India; b National Institute of Plant Genome Research, New Delhi, India

## Abstract

We report here the genome sequence of *Pandoraea* sp. ISTKB, a betaproteobacterium isolated from rhizospheric soil in the backwaters of Alappuzha, Kerala, India. The strain is alkalotolerant and grows on medium containing lignin as a sole carbon source. Genes and pathways related to lignin degradation were complemented by genomic analysis.

## GENOME ANNOUNCEMENT

Lignin is recalcitrant to degradation due to its complex and heterogeneous structure (1). Although industry based on plant polysaccharides will add to the already large amount of lignin generation (2), sustainable biorefinery can be possible if polysaccharides and lignin are utilized in improved ways. Fungi are well-known lignin degraders, but in recent years the focus has been on bacterial degradation, since bacteria are more adapted to extreme environments and are easy to manipulate at the genetic level (3, 4). *Pandoraea* sp. ISTKB is an alkalotolerant strain, and the degradation and decolorization potential of kraft lignin and various dyes have been investigated with this strain (5). Pretreatment of sugarcane bagasse by *Pandoraea* sp. ISTKB under submerged and solid state conditions was also studied in detail (6). Insight into genomic analysis might help us to understand the novel enzymes and pathways responsible for lignin degradation and biovalorization.

The draft genome of *Pandoraea* sp. ISTKB was sequenced using the Illumina MiSeq platform, which generated 2,139,250 paired-end reads of 151-bp length. Raw reads were filtered using the NGS toolkit version 2.3.1 to obtain 1,597,718 high-quality paired-end reads. The genome assembly was performed using Velvet version 1/2/10 (7), SOAPdenovo (8), and gsAssembler using a *k*-mer value of 57 for primary assembly. Scaffolding for the primary assembled contigs was performed using SSPACE (9) to generate 115 scaffolds, with an *N*_50_ scaffold size of 132,761 bp (maximum scaffold length: 545,170 bp; minimum scaffold length: 258 bp). Genes were identified using the NCBI Prokaryotic Genome Annotation Pipeline (PGAP). The functional annotation of the genome sequence was also performed using Pfam (10). Pathway analysis was carried out using the KEGG Automatic Annotation Server (KAAS) (11).

The total genome size of *Pandoraea* sp. ISTKB is 6.37 Mb, and a coverage of 65× was achieved. The strain had a G+C content of 62.05% and a total of 5,356 protein-coding genes were predicted. The bacterium contained 54 tRNAs, six rRNAs (two copies each for 5S, 16S, and 23S), and four ncRNAs. Additionally, 154 pseudogenes were also identified, out of which 12 were frame-shifted pseudogenes. With PGAP, 68.59% of the predicted proteins were annotated. In addition, Pfam annotation was assigned to 4,603 genes (85.94%), and the KAAS tool predicted 1,351 genes (25.22%) to be involved in different pathways.

The draft genome revealed the presence of putative genes responsible for the degradation of lignin and lignin-derived aromatic compounds. The lignin-degrading enzymes identified were DyP-type peroxidases, peroxidases, multicopper oxidases, esterases, coniferyl-alcohol dehydrogenase, coniferyl-aldehyde dehydrogenase, etherases, methyltransferases, and vanillate O-demethylase oxidoreductase. Genes responsible for the catabolism of aromatic compounds identified in the genome assembly were 4-hydroxybenzoate 3-monooxygenase, vanillate monooxygenase ferredoxin subunit, salicylate hydroxylase, protocatechuate 3,4-dioxygenase, gentisate 1,2-dioxygenase, catechol 2,3-dioxygenase, extradiol ring-cleavage dioxygenase, catechol 1,2-dioxygenase, phthalate 4,5-dioxygenase, protocatechuate 4,5-dioxygenase, hydroxyquinol 1,2-dioxygenase, 3-phenylpropionate dioxygenase, and various other oxidoreductases. Additionally, genes for oxidative stress and redox signaling, such as glutathione peroxidase, glutathione synthase, glutathione reductase, thioredoxin, thioredoxin reductase, catalases, and superoxide dismutases, were also present. These findings indicate that *Pandoraea* sp. ISTKB could potentially have an application in lignocellulosic biomass valorization.

### Accession number(s).

This whole-genome shotgun project has been deposited at DDBJ/ENA/GenBank under the accession number MAOS00000000. The version described in this paper is the first version, MAOS01000000.
